# Expression of insulin-like growth factor binding protein-3 in HELLP syndrome

**DOI:** 10.1186/s12884-023-06074-7

**Published:** 2023-11-10

**Authors:** Li Wei, Zhou Liping, Kang Suya

**Affiliations:** The Affiliated Suzhou Hospital of Nanjing Medical University, Suzhou Municipal Hospital, Gusu School, Nanjing Medical University, No. 26, Daoqian Street, Suzhou, Jiangsu China

**Keywords:** HELLP syndrome, Insulin-like growth factor binding protein-3 (IGFBP-3), Endothelial cell, Inflammatory response

## Abstract

**Objective:**

To investigate the expression of insulin-like growth factor binding protein-3(IGFBP-3) in HELLP syndrome and its possible role in the pathogenesis of this disease.

**Methods:**

1) 87 subjects were enrolled, including 29 patients with HELLP syndrome, 29 patients with pre-eclampsia (PE), and 29 healthy gravidae as control. The levels of IGFBP-3, IGF-1, TGF-β1, and VEGF in maternal and umbilical blood of them were detected using ELISA. Correlation analysis was used to observe the correlation between IGFBP-3 and IGF-1/TGF-β1/VEGF in maternal and umbilical blood, as well as that between maternal serum IGFBP-3 and clinical diagnostic indicators of HELLP syndrome. 2) Human hepatic sinusoid endothelial cells (HLSEC) and human umbilical vein endothelial cells (HUVEC) were cultured with different concentrations of IGFBP-3. After 72 h of culture, cell apoptosis and the normal living cells rate were detected and compared.

**Results:**

1) In both maternal and umbilical blood of HELLP group, levels of IGFBP-3 and TGF-β1 were higher than control and PE group, IGF-1was lower than control group, VEGF was lower than control and PE group. IGFBP-3 in maternal blood was correlated with IGF-1/TGF-β1/ VEGF, while IGFBP-3 in umbilical blood was linked to IGF-1/TGF-β1. In maternal blood, there was a negative correlation between PLT and IGFBP-3, and a positive correlation between ALT/AST/LDH and IGFBP-3. 2) After cultured with IGFBP-3, the total apoptosis rate of either HLSEC or HUVEC was considerably elevated, while the normal living rate was decreased.

**Conclusion:**

The expression of IGFBP-3 is elevated in HELLP syndrome, which may subsequently promote cell apoptosis by affecting the expression and function of IGF-1, VEGF, and TGFβ1 in the IGF/PI3K/Akt, TGF-β1/Smad3, and VEGF/eNOS/NO pathways. IGFBP-3 aggravates inflammatory reactions of the vascular endothelium and liver under hypoxia, affects the normal function of cells, and plays a role in the pathogenesis of diseases.

Hemolysis, elevated liver enzymes, and low platelet count (HELLP) syndrome is a severe complication of pregnancy, which often involves multiple organs and various systems of the maternal body [1]. The incidence rate of HELLP syndrome in pregnancy is about 0.5–0.9%, besides, its incidence rate in pre-eclampsia (PE) cases is approximately 10–20% [2]. Although occurs infrequently, it can cause serious maternal complications including disseminated intravascular coagulation (DIC), acute renal failure, and hepatic subcapsular hemorrhage [3, 4]. It may also lead to conditions that affect the fetus, such as preterm birth, fetal growth restriction, and fetal intrauterine distress [5–7]. It has been reported that the maternal mortality rate of HELLP syndrome is about 1%-24%, while the perinatal mortality rate varies between 6.7 and 70% [8].

Clinical diagnosis of the disease is mainly based on laboratory examination results [9, 10]. However, there is still a lack of indicators for early diagnosis and prediction. Currently, it is believed that the most effective treatment for HELLP syndrome is the termination of pregnancy [11, 12]. Other therapies such as glucocorticoids and plasmapheresis are mainly symptomatic treatments [13–20]. So far, there are no effective targeted measures to halt the progression of HELLP syndrome, let alone etiotropic measures to prevent its occurrence [21]. Early diagnosis and timely treatment are of great importance in reducing the adverse outcomes of HELLP syndrome. Thus, it is an urgent issue to explore the pathogenesis of HELLP syndrome, discover efficient indicators in diagnosis, prediction, and prognosis, and find additional targets for therapy.

Most researchers agree that HELLP syndrome is similar to PE in its main pathological changes. However, it produces a more intense inflammatory response, which tends to damage the liver and clotting system [22]. Although the exact etiology is uncertain, it is believed that the disease may be caused by the interaction of multiple mechanisms under the influence of various factors. Some studies have asserted that the damage to vascular endothelial cells and hepatic sinusoidal endothelial cells is the key link in the pathophysiological mechanism of HELLP syndrome [23, 24]. Etiology research about it has focused on genetics, immunology, and lipid metabolism, as well as other aspects. Specially, the role of the excessive inflammatory response caused by inappropriate activation of certain cytokines should not be ignored [25–38]. A variety of factors, such as ischemia and hypoxia, may upset the balance of the maternal body during pregnancy, leading to inappropriate activation of relevant cytokines and resulting in endothelial dysfunction and a series of pathophysiological changes. These factors may be crucial in the progression of HELLP syndrome [39, 40].

In our previous study, to explore the pathogenesis of HELLP syndrome, protein microarray analysis was performed on the sera of patients with HELLP syndrome and healthy pregnant women. In this analysis, 30 proteins with distinctly different expressions were isolated. Further bioinformatics analysis revealed that seven of them were closely related to the disease, including insulin-like growth factor binding protein-3 (IGFBP-3) [41]. The insulin-like growth factor binding protein (IGFBP) is a superfamily of proteins that specifically binds to insulin-like growth factor (IGF). Besides, IGFBP-3, a member of the family, most of which is produced in the placenta and liver [42]. It plays a role in the regulation of multiple biomolecular signaling pathways through IGF-dependent and IGF-independent pathways and regulates cell growth, differentiation, apoptosis, and other physiological processes [43–46]. Also, IGFBP-3is closely related to non-infectious liver inflammation, malignant tumors, and other diseases [47–50], as well as pregnancy-related diseases such as fetal growth restriction and gestational diabetes mellitus [51, 52].

HELLP syndrome is viewed as a disease of placental origin as PE, pathophysiological changes of which secondary to the dysfunction of placenta-endothelium-liver axis play an important role in promoting the course of the disease. The superficial invasion of the trophoblast into the myometrium affects the recasting of the uterine spiral artery, resulting in ischemia, hypoxia, and abnormal function of the placenta. Subsequently, a variety of placental factors are released, thereby activating endothelial cells and leading to injury [32–35, 53]. The above process causes a strong inflammatory response of vascular endothelium and releases inflammatory mediators with hepatotoxicity, reaching the liver through blood circulation and leading to the injury of hepatic sinusoid endothelium cells. Subsequently, sinusoid obstruction syndrome (SOS) is induced, sometimes eventually causing liver failure. This process is an important link in the pathogenesis of HELLP syndrome [54–59]. There are various placenta-derived factors involved in the placenta-endothelium-liver axis, such as necrotic fragments and antiangiogenic factors, as well as IGFBP-3 [54].

To date, IGFBP-3 has not yet been studied in HELLP syndrome. Based on the results of our previous research and the existing known pathogenesis of HELLP syndrome, a speculation has been made. Through the inhibition of the IGF/PI3K/Akt pathway, activation of the TGF-β1/Smad3 pathway, and alteration of the vascular endothelial growth factor (VEGF) function, IGFBP-3 may reduce the generation of nitric oxide (NO) and induce the release of inflammatory factors. It may also strengthen the inflammatory response of vascular endothelial cells and hepatic sinusoid endothelial cells and promotes the apoptosis of cells, thus playing a role in the damage to endothelial cells and the liver in HELLP syndrome.

Through experimental verification in clinical specimens and cells, we carried out research on the expression of IGFBP-3 in HELLP syndrome and its clinical significance in this study. Also, we assessed its possible role in the disease mechanism, aiming to find new serum markers of HELLP syndrome and provide approaches for reducing the harm to mothers and children caused by the disease.

## Materials and methods

### Patients and samples

The sample size was calculated as *α* (test level) = 0.05, 1-*β* (test performance) = 0.9, using PASS 15.0 software(NCSS, Kaysville, Utah, USA). The sample size estimator of *ANOVA* for multi-group mean comparison was selected, and the corresponding increase of 15% was combined with the requirement of non-parametric test. The total sample size required for the three groups was estimated to be about 78 cases. In this study, a total of 87 pregnant women who gave birth at the Suzhou Affiliated Hospital of Nanjing Medical University /Suzhou Municipal Hospital from January 2018 to June 2020 participated in this study. The cohort included 29 patients with HELLP syndrome, 29 patients with PE but without HELLP syndrome, and 29 healthy pregnant women as controls. The groups were named as follows: ①HELLP group; ②Pre-eclampsia group (PE group); ③ Control group.

The inclusion criteria of the HELLP group followed the Tennessee diagnostic criteria [9]: platelets (PLT) < 150 × 10^9^/L, elevated lactate dehydrogenase (LDH) > 600 IU/L, aspartate aminotransferase (AST) and/or alanine aminotransferase (ALT) ≥ 70U/L. Besides, patients were divided into Class I ~ III based on their platelet count according to the Mississippi classification [10]: Class I: < 50 × 10^9^/L; Class II: 50–100 × 10^9^/L; Class III: 100–150 × 10^9^/L. The selected patients suffered from complete HELLP syndrome, including ten of Class I, 17 of Class II, and two of Class III. The diagnostic criteria of PE were in accordance with Gestational Hypertension and Pre-eclampsia: ACOG Practice Bulletin, published in 2020 [9].

The exclusion criteria were as follows: ①Accompanied by complications caused by other liver dysfunctions, such as viral hepatitis, intrahepatic cholestasis during pregnancy, acute fatty liver during pregnancy, etc.;②Other thrombocytopenic complications, such as thrombocytopenia in pregnancy, hemolytic uremia in pregnancy, etc.;③Pregnant women with a history of inflammation and infection.

Peripheral venous blood samples were collected from all pregnant women within 24 h of admission, while patient samples were collected before the initiation of any medical treatment. Next, 2 mL blood was placed into EDTA tubes, left to stand for 10 min, then centrifuged at 3000 rpm for 10 min. The sera were then separated, packaged, and cryopreserved at -80 ℃ for subsequent experiments. Samples of umbilical blood were collected during delivery, then separated and preserved in the same way as above.

Demographic characteristics and clinical information of all participants were recorded, including age, gravidity, parity, sampling time, gestational time, and neonatal weight, as well as the primary laboratory indicators related to the diagnosis of HELLP syndrome.

All pregnant women involved provided informed consent to participate in this study, which was reviewed and approved by the Ethics Committee of the Suzhou Affiliated Hospital of Nanjing Medical University.

### Reagents

The reagents comprised: Human IGFBP-3 ELISA kit (R&B, DY675, USA), IGF-1 ELISA kit (R&B, DY291-05, USA), TGF-β1 ELISA kit (R&B, DY240-5,USA), Human VEGF ELISA Kit (R&B, DY293B-5,USA), goat serum (Proteintech Inc., B900780, USA), Tween-20 (Sigma Inc., T2700, USA), substrate solution (R&B,DY999, USA), bovine serum albumin (Sigma, B2064-50,USA), Annexin V-FITC/PI apoptosis assay kit (Novizan, A211-01, China), endothelial cell culture medium (ScienCell, 1001, USA), recombinant human IGFBP-3 protein (YiqiaoShenzhou Technology Co., Ltd., 10430-H07H, China).

### Instruments

The equipment used in this study included: flow cytometer (Gallios, Beckman), cryogenic high-speed centrifuge (Sorvall LYNX 4000, Thermo Fisher), SX-BC-1000A2 biosafety cabinet(SujinAntai), 31L1 water capsule CO2 cell incubator (Thermo Fisher), Bio-Plex 200 system petri dish (Bio-RAD Laboratories, Inc.), multi-mode microplate reader (Molecular Devices), tabletop centrifuge (Beckman Coulter).

### Cell lines

Human liver sinusoidal endothelial cells (HLSEC) and human umbilical vein endothelial cells (HUVEC) were purchased from ATCC. All cells were genetically identified by short tandem repeat (STR) analysis and confirmed to be free of mycoplasma infection.

### Methods

#### Enzyme-Linked Immunosorbent Assay (ELISA)

The concentrations of IGFBP-3, IGF-1, TGF-β1, and VEGF in maternal and umbilical blood of each group were determined according to the operating procedure of the ELISA kit instructions.

#### HLSEC and HUVEC culture

HLSEC and HUVEC were cultured in an ECM medium containing 10% FBS in 6-well plates (5 × 10^5^ cells/well) in an incubator containing 5%CO_2_ at 37 ℃. After cell convergence reached 60–70%, IGFBP-3 with gradients of 0 nM (control group), 10 nM (10 nM group), 20 nM (20 nM group), and 40 nM (40 nM group) were added. Apoptosis of cells was detected after 72 h of culture.

#### Apoptosis detection

The cells were digested by trypsin without EDTA and terminated by medium. The digested suspension was then centrifuged at 300 g and 4 ℃ for 5 min. Cells were collected and rinsed three times with pre-cooled cell staining buffer. The tubes were re-suspended with 100μL Annexin V binding buffer and gently mixed with 5µL FITC Annexin V and 10 µL propidium iodide staining solution, then incubated for 15 min at room temperature away from light. Next, 400 µL Annexin V binding buffer was added to the test tube and mixed, then the cell apoptosis index was determined by flow cytometry.

#### Correlation analysis

To determine whether changes in IGF-1, TGF-β1, and VEGF correlated with variations in IGFBP-3 serum levels, we analyzed the associations between the IGFBP-3results in maternal blood and umbilical blood with these three indicators for each group. Once the correlation was confirmed, further linear regression analysis was performed to determine whether the correlation was linear.

### Statistical analysis

SPSS Statistics for Windows, Version 22.0 (IBM, Chicago, IL, USA) was used for statistical analysis. Besides, the *K-S* test was applied for quantitative data and mean ± standard error of the mean [*Mean* ± *SEM*; ($\overline{x }\pm s$)] was used for data subject to a normal distribution. The independent sample *T* test was employed for comparisons between two groups, while the one-way *ANOVA* test was used for comparisons among three groups. The homogeneity test of variance was performed first, then a pairwise comparison was conducted using *LSD* for homogenous groups, otherwise, *Dunnett’s T3* test was used. Data that did not follow normal distribution were expressed by a median (percentile) [*M* (*P*_25_, *P*_*75*_)] and compared using the *Mann-Whitney U* test. Qualitative data were expressed as a percentage (%), the*χ2* test was employed for comparisons between two groups, the *row* × *columnχ2* test was used for comparisons among three groups, and the *Bonferroni* test was applied for further analysis between every two groups. Next, the *Pearson* or *Spearman* analysis was used to determine whether there were correlations between two groups of continuous data. When both groups of continuous data followed a normal distribution, the *Pearson* correlation analysis was employed, otherwise, the *Spearman* analysis was used. Absolute values of the correlation coefficient *r* above 0.7 indicated a strong association, 0.4 to 0.7 implied a moderate correlation, and 0.2 to 0.4 suggested a weak connection. Besides, values of *P* < 0.05 suggested statistical significance.

## Results

### Fundamental states of pregnant women in each group

Comparisons based on demographic characteristics and main laboratory indicators of all subjects among the three groups are shown in Table 1.

**Table 1:** Comparison of clinical information among three groups

**Groups**	**① HELLP** (*n* = 29)	**② PE** (*n* = 29)	**③ Control** (*n* = 29)	***F/H/χ2***	***P***
**Age (years)**	30.76 ± 5.12	30.69 ± 4.31	28.97 ± 4.20	1.436	0.244
**Gravidity**	2.00 (1.00, 3.00)	2.00 (1.00, 3.00)	2.00 (2.00, 3.00)	0.788	0.674
**Parity**	1.00 (1.00, 2.00)	2.00 (1.00, 2.00)	2.00 (1.00, 2.00)	0.413	0.813
**Gestational age of sampling (weeks)**	33.62 ± 3.56	33.63 ± 3.87	38.89 ± 1.27	27.519	0.000^**^
				①:②	0.991^**^
				①:③	0.000^**^
				②:③	0.000^**^
**Gestational age of delivery (weeks)**	33.93 ± 3.44	34.78 ± 3.90	39.63 ± 0.99	29.279	0.000^**^
				①:②	0.760
				①:③	0.000^**^
				②:③	0.000^**^
**Rate of SGA % (n)**	51.7 (15)a	44.8 (13)a	6.9 (2)b	14.958	0.001^**^
**Neonatal weight (g)**	1974.66 ± 820.78	2187.24 ± 879.51	3451.72 ± 406.53	34.403	0.000^**^
				①:②	0.715
				①:③	0.000^**^
				②:③	0.000^**^
**Systolic pressure (mmHg)**	161.00 (153.50, 176.50)	149.00 (141.50, 161.50)	118.00 (113.50, 126.50)	60.031	0.000^**^
				①:②	0.110
				①:③	0.000^**^
				②:③	0.000^**^
**Diastolic pressure (mmHg)**	107.00 (99.00, 114.00)	97.00 (86.00, 102.00)	75.00 (69.00, 80.00)	58.339	0.000^**^
				①:②	0.051
				①:③	0.000^**^
				②:③	0.000^**^
**Mean arterial pressure (mmHg)**	123.33 (116.83, 133.83)	113.33 (104.17, 120.67)	89.67 (84.00, 94.50)	61.227	0.000^**^
				①:②	0.066
				①:③	0.000^**^
				②:③	0.000^**^
**PLT (10**^**9**^**/L)**	62.00 (44.00, 80.50)	179.00 (154.50, 209.50)	180.00 (143.50, 241.50)	55.662	0.000^**^
				①:②	0.000^**^
				①:③	0.000^**^
				②:③	1.000
**ALT (U/L)**	104.00 (62.00, 255.50)	21.00 (16.50, 28.50)	9.00 (7.00, 12.50)	69.653	0.000^**^
				①:②	0.000^**^
				①:③	0.000^**^
				②:③	0.000^**^
**AST (U/L)**	116.00 (61.00, 252.00)	20.00 (17.50, 25.50)	19.00 (15.003, 21.50)	53.915	0.000^**^
				①:②	0.000^**^
				①:③	0.000^**^
				②:③	0.700
**LDH (U/L)**	1470.00 (950.50, 2206.50)	447.00 (399.00, 542.00)	203.00 (177.50, 266.00)	72.687	0.000^**^
				①:②	0.000^**^
				①:③	0.000^**^
				②:③	0.000^**^
**Bilirubin (mg/L)**	10.80 (5.90, 19.05)	4.30 (1.10, 7.25)	8.80 (6.60, 11.95)	23.665	0.000^**^
				①:②	0.000^**^
				①:③	1.000
				②:③	0.000
**Scr (μmol/L)**	73.00 (62.15, 97.90)	52.00 (47.00, 58.50)	45.00 (43.00, 50.45)	43.933	0.000^**^
				①:②	0.000^**^
				①:③	0.000^**^
				②:③	0.078
**BUN (mmol/L)**	6.86 (5.38, 8.94)	3.93 (3.30, 4.74)	3.54 (2.64, 4.26)	46.721	0.000^**^
				①:②	0.000^**^
				①:③	0.000^**^
				②:③	0.153

*ALT* Alanine transaminase, *AST* Aspartate aminotransferase, *LDH* Lactate dehydrogenase, *Scr* Serum creatinine, *BUN* Blood urea nitrogen

^*^P < 0.05, ^**^P < 0.01, a-b: *P* < 0.05

There were no significant differences in terms of age, parity, and gravidity among the three groups (all *P* > 0.05). Besides, there were no statistically significant differences in terms of gestational age (both of sampling and delivery)and neonatal weight between the PE group and the HELLP group (both *P* > 0.05), while the figures in the control group were both higher than the other two groups (all *P* < 0.01). The rate of SGA (small for gestational age infant) between the PE group and the HELLP group didn’t show significant difference (*P* > 0.05), while the rate were both higher than that of the control group (both *P* < 0.05) [60, 61]. Additionally, there were no statistically significant differences in terms of systolic pressure, diastolic pressure, and mean arterial pressure between the PE group and the HELLP group (all* P* > 0.05). However, the values in these two groups were all significantly higher than the control group (all *P* < 0.01).

The primary laboratory indicators related to the diagnosis of HELLP syndrome were compared as follows: the PLT in the HELLP group was significantly lower than that in the control and PE groups (both *P* < 0.01). However, there was no significant difference between the control group and the PE group (*P* > 0.05). ALT levels were highest in the HELLP group, followed by the PE group and then the control group, and there were significant differences between each pair of groups (all *P* < 0.01). AST levels in the HELLP group was noticeably higher than in the control group and the PE group (both *P* < 0.01). However, the difference between the control group and the PE group was negligible (*P* > 0.05). Levels of LDH were highest in the HELLP group, followed by the PE group and then the control group. Besides, the differences between each group were considerable (all *P* < 0.01). Concerning serum bilirubin, levels in the PE group were lower than both of the other two groups (*P* < 0.01), while there was a negligible difference between the control group and the HELLP group (*P* > 0.05).

In terms of biochemical indexes related to renal function, including serum creatinine and urea nitrogen, levels in the HELLP group was significantly higher than in the control group and the PE group (*P* < 0.01). However, there was no significant difference between the control group and the PE group (*P* > 0.05).

### Levels of IGFBP-3, IGF-1, TGF-β1, and VEGF in maternal and umbilical blood

#### Levels of IGFBP-3, IGF-1, TGF-β1, and VEGF in maternal blood

**Fig. 1:**
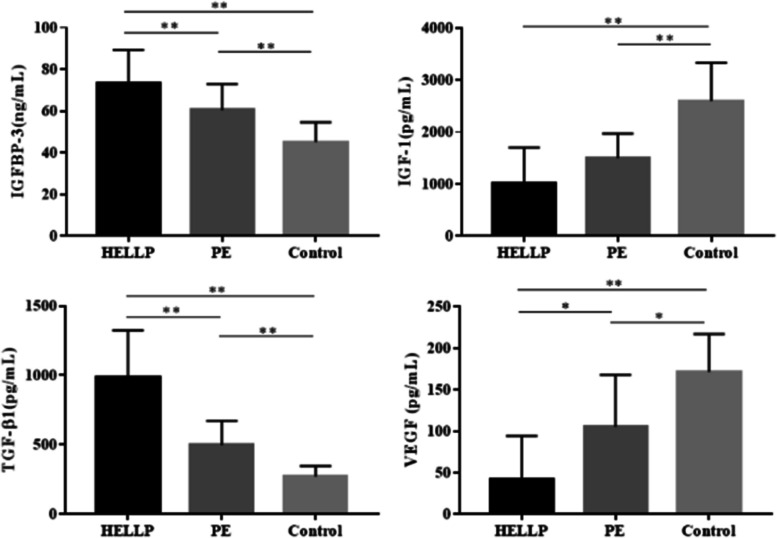
Levels of IGFBP-3/IGF-1/TGF-β1/VEGF in maternal blood of three groups (^*^P < 0.05, ^**^P < 0.01)

The results of these tests, which are presented in Table 2 and Fig. 1, show that the levels of IGFBP-3 in the maternal blood of the HELLP group were the highest, while those of the PE group were also higher than the control group. Besides, the differences among the three groups and between each pair of groups were statistically significant (all *P* < 0.01).

**Table 2:** Levels of IGFBP-3/IGF-1/TGF-β1/VEGF in maternal blood of three groups

**Indicators in maternal blood**	**① HELLP 组** (*n* = 29)	**② PE 组** (*n* = 29)	**③ Control 组** (*n* = 29)	***F/H***	***P***
**IGFBP-3 (ng/mL)**	58.15 ± 15.95	45.42 ± 16.54	36.62 ± 9.10	35.22	0.000^**^
				①:②	0.003^**^
				①:③	0.000^**^
				②:③	0.000^**^
**IGF-1 (pg/mL)**	1019.05 (367.05, 1700.73)	1497.16 (739.42, 1969.28)	2597.90 (2153.57, 3333.22)	39.80	0.000^**^
				①:②	0.263
				①:③	0.000^**^
				②:③	0.000^**^
**TGF-β1 (pg/mL)**	982.00 ± 342.81	495.65 ± 176.50	270.87 ± 74.53	74.535	0.000^**^
				①:②	0.000^**^
				①:③	0.000^**^
				②:③	0.000^**^
**VEGF (pg/mL)**	42.11 (19.95, 94.33)	105.59 (37.22, 167.75)	171.28 (92.78, 217.28)	25.179	0.000^**^
				①:②	0.027^*^
				①:③	0.000^**^
				②:③	0.048^*^

^*^P < 0.05, ^**^P < 0.01

The levels of IGF-1 in the maternal blood of the HELLP group and the PE group were significantly lower than in the control group (*P* < 0.01). In contrast, there was a negligible difference between the HELLP group and the PE group (*P* > 0.05).

In the HELLP group, the levels of TGF-β1 in maternal blood were the highest, followed by the PE group and the control group. The overall difference among the three groups, as well as the pairwise comparisons between groups, were statistically significant (all* P* < 0.01).

The levels of VEGF in maternal blood were the lowest in the HELLP group, while those were the highest in the control group. The overall difference among the three groups was statistically significant (*P* < 0.01), while pairwise comparisons showed that VEGF levels in the HELLP group were lower than in the control group (*P* < 0.01) and the PE group (*P* < 0.05). Also, VEGF levels in the PE group were lower than in the control group (*P* < 0.05).

### Levels of IGFBP-3, IGF-1, TGF-β1, and VEGF in umbilical blood

**Fig. 2:**
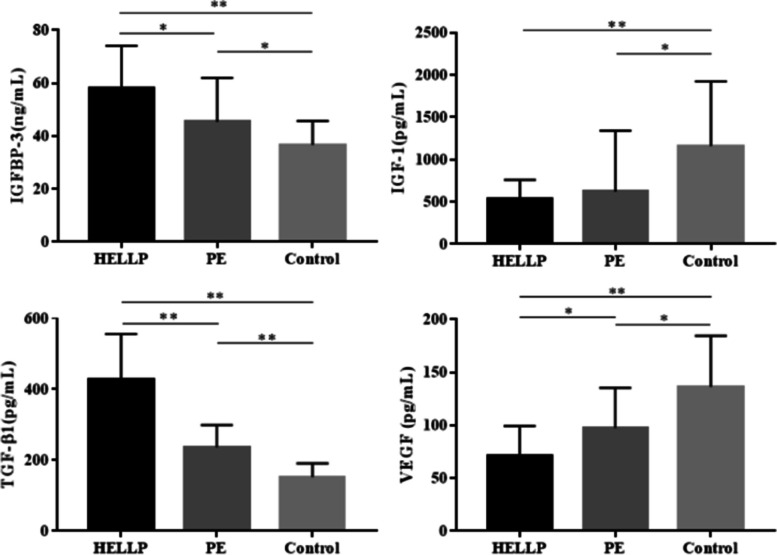
Levels of IGFBP-3/IGF-1/TGF-β1/VEGF in umbilical blood of three groups (^*^P < 0.05, ^**^P < 0.01)

The results of levels in umbilical blood are displayed in Table 3 and Fig. 2. The levels of IGFBP-3 in umbilical blood varied considerably among the three groups (*P* < 0.01). IGFBP-3 levels in the HELLP group were higher than both the control group (*P* < 0.01) and the PE group (*P* < 0.05), and those of the PE group were also higher than the control group (*P* < 0.05).

**Table 3:** Levels of IGFBP-3/IGF-1/TGF-β1/VEGF in umbilical cord blood of three groups

**Indicators in umbilical blood**	**① HELLP** (*n* = 29)	**② PE** (*n* = 29)	**③ Control** (*n* = 29)	***F/H***	***P***
**IGFBP-3 (ng/mL)**	58.15 ± 15.95	45.42 ± 16.54	36.62 ± 9.10	16.68	0.000^**^
				①:②	0.047^*^
				①:③	0.000^**^
				②:③	0.013^*^
**IGF-1 (pg/mL)**	533.70 (483.62, 789.89)	622.19 (480.27, 1339.70)	1159.29 (681.83, 1923.70)	19.956	0.000^**^
				①:②	0.185
				①:③	0.000^**^
				②:③	0.030^*^
**TGF-β1 (pg/mL)**	426.60 ± 129.80	236.65 ± 62.89	152.40 ± 38.37	77.047	0.000^**^
				①:②	0.000^**^
				①:③	0.000^**^
				②:③	0.000^**^
**VEGF (pg/mL)**	71.04 (49.11, 99.24)	97.43 (71.57, 135.24)	136.32 (101.80, 184.42)	24.340	0.000^**^
				①:②	0.041^*^
				①:③	0.000^**^
				②:③	0.023^*^

^*^P < 0.05, ^**^P < 0.01

The level of IGF-1 in umbilical blood were noticeably different among the three groups (*P* < 0.01). Those of the control group were higher than both the HELLP group (*P* < 0.01) and the PE group (*P* < 0.05), although the difference between the HELLP group and the PE group was not statistically significant (*P* > 0.05).

The levels of TGF-β1 in umbilical blood also varied substantially among the three groups (*P* < 0.01). Levels in the HELLP group were higher than the control group and the PE group, and those of the PE group were higher than the control group (all *P* < 0.01).

VEGF levels in umbilical blood exhibited statistically significant differences among the three groups (*P* < 0.01). Levels in the HELLP group were lower than the control group (*P* < 0.01) and the PE group (*P* < 0.05), while those of the PE group were lower than the control group (*P* < 0.05).

### Results of correlation analysis between IGFBP-3 and IGF-1/TGF-β1/VEGF

**Fig. 3:**
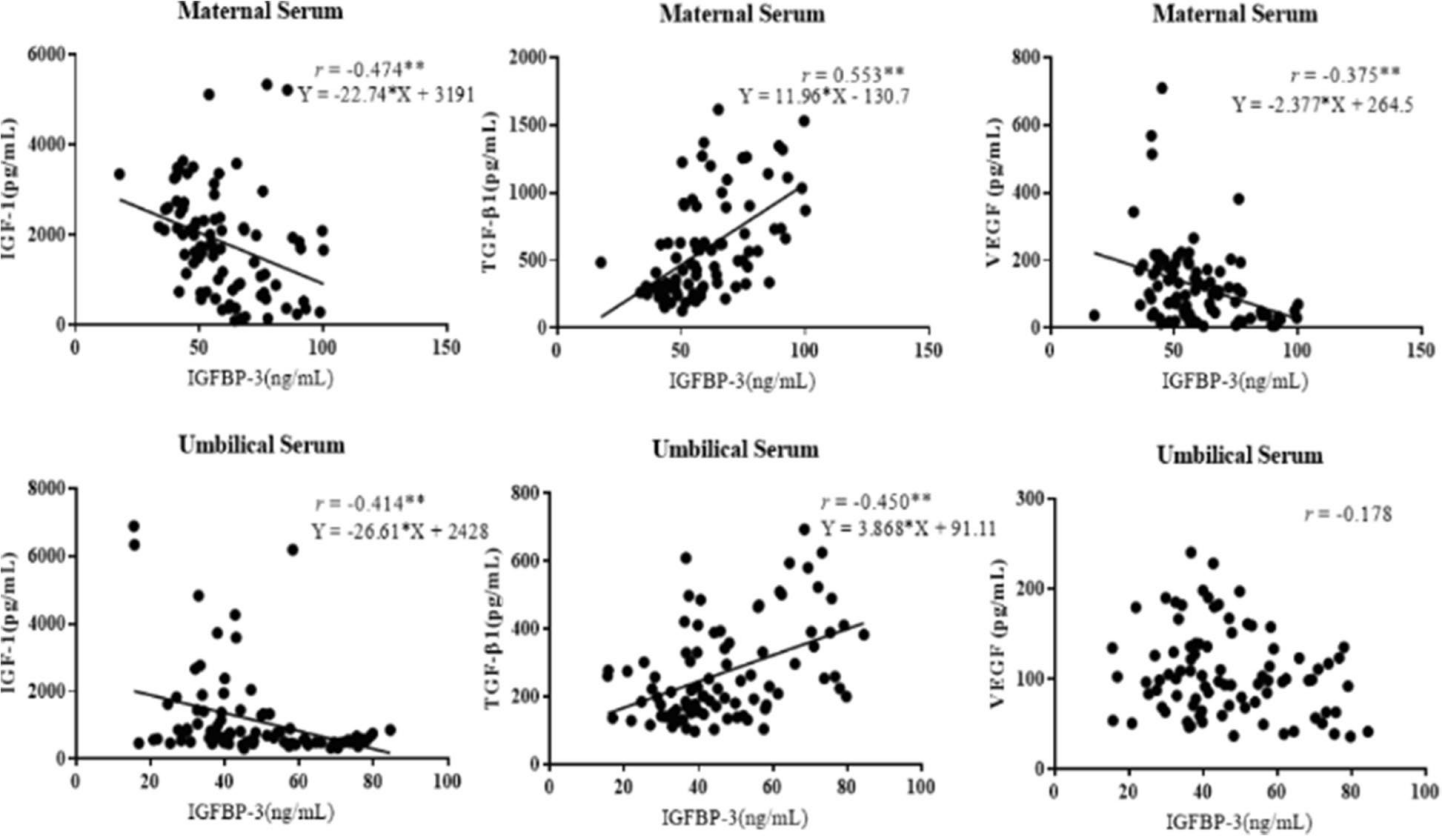
Correlation analysis between IGFBP-3 and IGF-1/TGF-β1/VEGF (^**^P < 0.01, Line 1: results of correlation analysis in maternal blood; Line 2: results of correlation analysis in umbilical blood. The correlation coefficient (*r*) and regression equation are shown in the figure

As shown in Fig. 3, the levels of IGF-1 in maternal blood were negatively correlated with IGFBP-3(*r* = -0.474). Besides, the levels of TGF-β1 were positively correlated with IGFBP-3 (*r* = 0.553); VEGF expression were negatively correlated with IGFBP-3 (*r* = -0.375). The results described above all presented statistically significant differences (all *P* < 0.01). Further linear regression analysis revealed that there were linear correlations between IGFBP-3 and IGF-1/TGF-β1/VEGF in maternal blood (all *P* < 0.01).

The levels of IGF-1 in umbilical blood were negatively correlated with IGFBP-3 (*r* = -0.414). Additionally, TGF-β1 levels were positively correlated with IGFBP-3 (*r* = 0.450). Both of the above correlations exhibited statistical significance (*P* < 0.01). However, although VEGF levels were negatively correlated with IGFBP-3 (*r* = -0.178), the results did not exhibit statistical significance (*P* > 0.05). Further regression analysis showed that there were significant linear correlations between IGFBP-3 and IGF-1/TGF-β1 (both *P* < 0.01).

### Results of correlation analysis between IGFBP-3 in maternal blood and main diagnostic indexes of HELLP syndrome

**Fig. 4:**
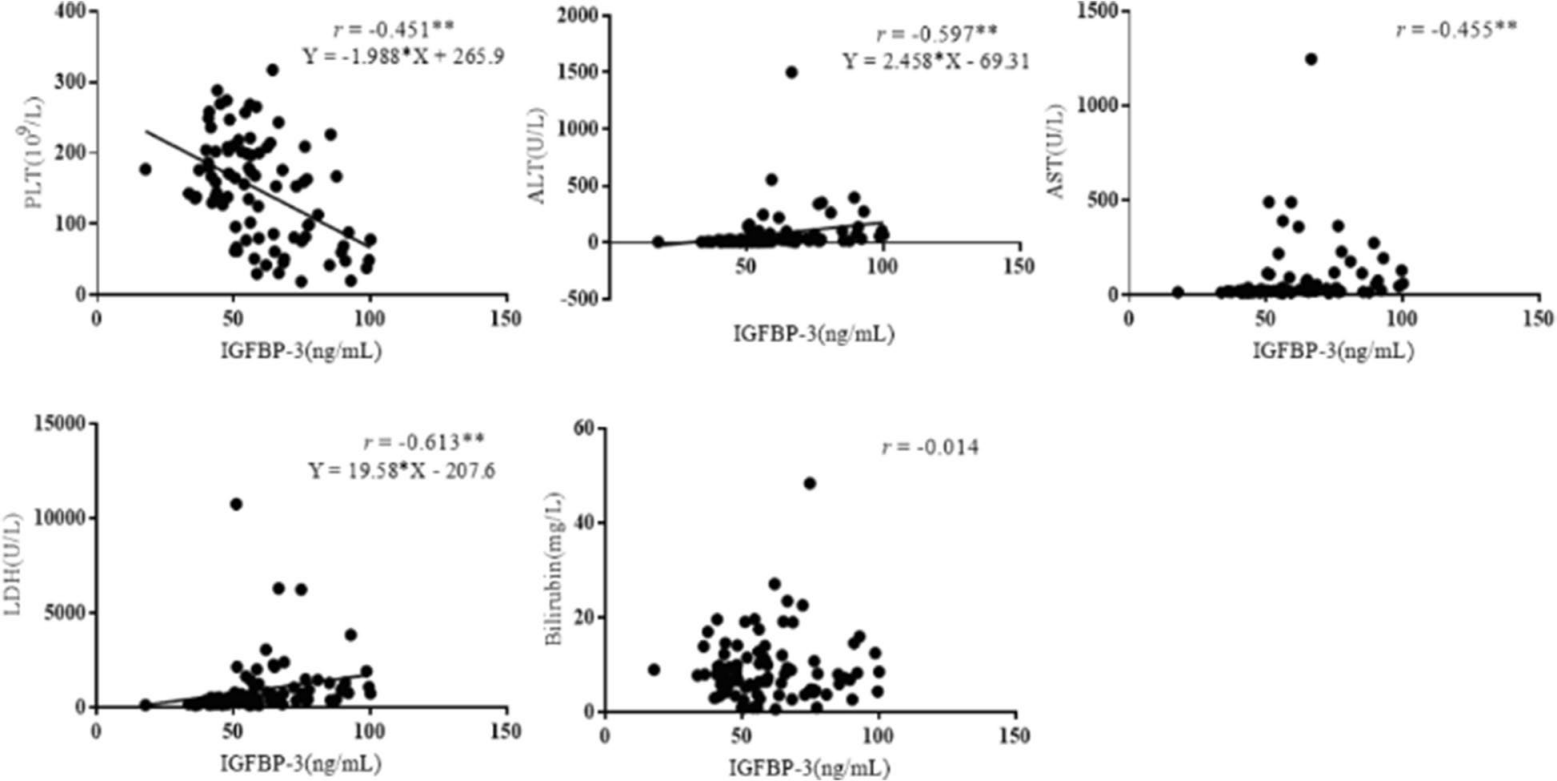
Correlation analysis between IGFBP-3 and diagnostic parameters of HELLP syndrome (^**^P < 0.01)

As shown in Fig. 4, there was a moderate negative correlation between peripheral blood platelet count and the level of IGFBP-3 in pregnant women (*r* = -0.451), with a statistically significant difference (*P* < 0.01).

Moreover, there was a moderate positive correlation between the serum ALT and IGFBP-3 of pregnant women (*r* = 0.597, *P* < 0.01), while a regression analysis showed that there was a linear correlation between the two indices (*P* < 0.01). There was also a positive correlation between the serum AST and IGFBP-3 of pregnant women (*r* = 0.455, *P* < 0.01). However, no significant linear correlation was found between these two indices (*P* > 0.05).

There was a moderate positive correlation between the serum LDH and IGFBP-3 of pregnant women (*r* = 0.613, *P* < 0.01), with a significant linear correlation (*P* < 0.01). However, there was not a significant correlation between the serum bilirubin and IGFBP-3 of pregnant women (*r* = -0.014, *P* > 0.05).

### Effect of IGFBP-3 on apoptosis of HLSEC and HUVEC

#### Effect of IGFBP-3 on HLSEC apoptosis

**Fig. 5:**
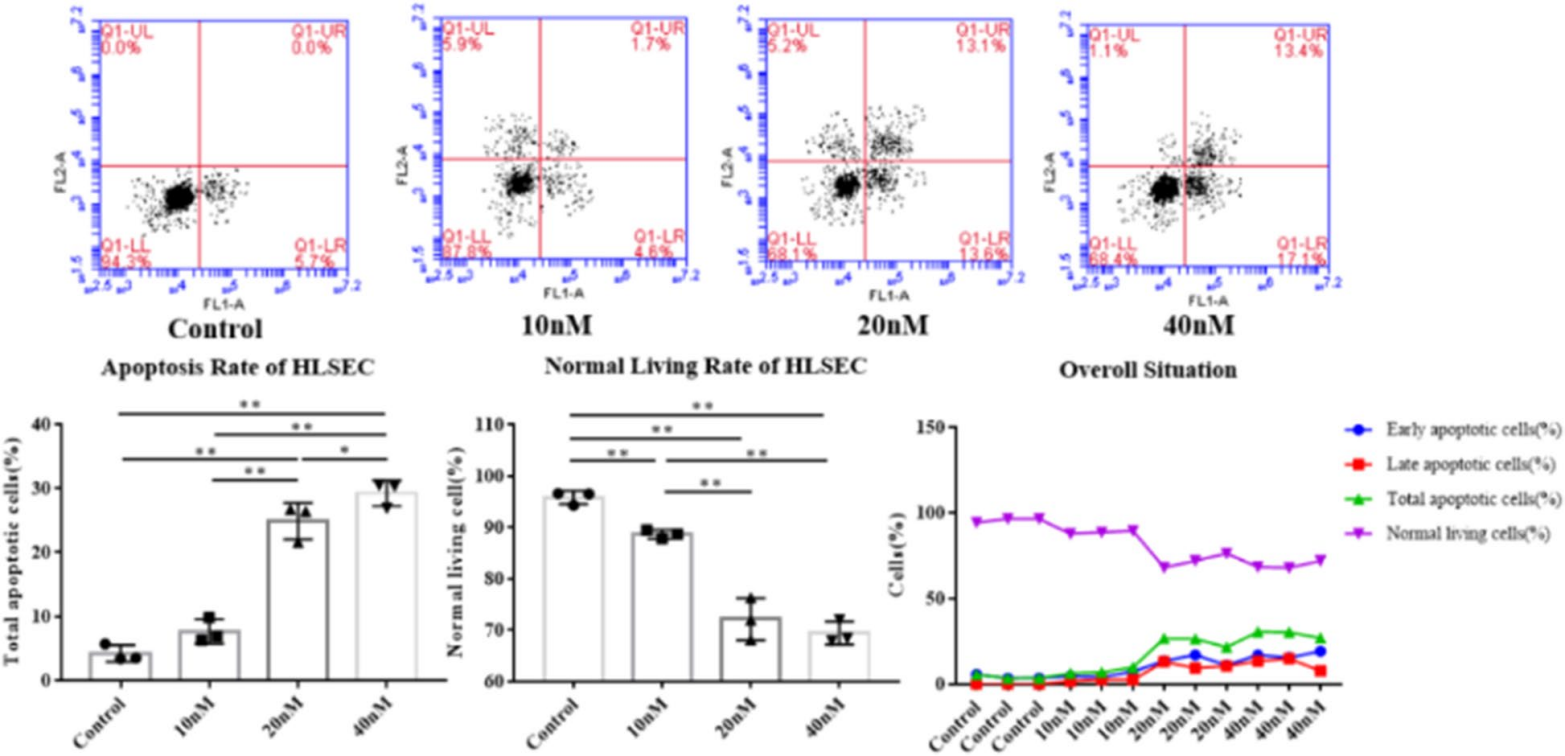
Comparison of apoptosis rate and normal living rate of HLSEC in each group (^*^P < 0.05, ^**^P < 0.01)

As Table 4 and Fig. 5 illustrate, the total apoptosis rate (%) of HLSEC cultured in different concentrations of IGFBP-3 was 4.20 ± 1.30 in the control group, 7.67 ± 1.87 in the 10 nM group, 24.90 ± 2.86 in the 20 nM group, and 29.23 ± 2.02 in the 40 nM group. There were significant statistical differences among the four groups (*P* < 0.01), except for the difference between the control group and the 10 nM group (*P* > 0.05). The total apoptosis rate in the 40 nM group was significantly higher than any of the other groups (all *P* < 0.01), while that of the 20 nM group was higher than both the control group and the 10 nM group (both *P* < 0.01).

**Table 4:** Apoptosis of two kinds of endothelial cells (HLSEC&HUVEC) cultured with different concentrations of IGFBP-3

**Groups**		**① Control** (*n* = 3)	**② 10 nM** (*n* = 3)	**③ 20 nM** (*n* = 3)	**③ 40 nM** (*n* = 3)	***F/H***	***P***
**HLSEC**	**Total rate of apoptosis (%)**	4.20 ± 1.30	7.67 ± 1.87	24.90 ± 2.86	29.23 ± 2.02	105.745	0.000^**^
						①:②	0.077
						①:③	0.000^**^
						①:④	0.000^**^
						②:③	0.000^**^
						②:④	0.000^**^
						③:④	0.000^**^
	**Normal living rate (%)**	95.80 ± 1.30	88.67 ± 0.85	72.13 ± 4.10	69.43 ± 2.24	80.723	0.000^**^
						①:②	0.008^**^
						①:③	0.000^**^
						①:④	0.000^**^
						②:③	0.000^**^
						②:④	0.000^**^
						③:④	0.216
**HUVEC**	**Total rate of apoptosis (%)**	3.90 ± 0.80	13.30 ± 3.47	26.00 ± 1.57	33.93 ± 4.28	63.652	0.000^**^
						①:②	0.004^**^
						①:③	0.000^**^
						①:④	0.000^**^
						②:③	0.001^**^
						②:④	0.000^**^
						③:④	0.010^*^
	**Normal living rate (%)**	96.10 (95.70, 96.5)	83.8 (82.20, 86.35)	73.30 (70.60, 73.3)	62.1 (60.10, 65.10)	10.009	0.018^*^
						①:②	1.000
						①:③	0.187
						①:④	0.019^*^
						②:③	1.000
						②:④	0.323
						③:④	1.000

^*^P < 0.05, ^**^P < 0.01

The normal living cell rate(%) of HLSEC was 95.80 ± 1.30 in the control group, 88.67 ± 0.85 in the 10 nM group, 72.13 ± 4.10 in the 20 nM group, and 69.43 ± 2.24 in 40 nM group, with significant differences among the four groups(*P* < 0.01). Comparisons between each pair of groups also exhibited considerable differences, except that between the 20 nM group and the 40 nM group (*P* > 0.05). The normal living cell rate in the 40 nM group was substantially lower than both the control group and the 10 nM group (both *P* < 0.01), while that of the 20 nM group was lower than the control group and the 10 nM group (both *P* < 0.01). Also, the rate of the 10 nM group was higher than the control group (*P* < 0.01).

These results reveal that IGFBP-3 increases the total apoptosis rate of HLSEC, which grows with rising IGFBP-3 concentrations. However, it reduces the normal living cell rate, which falls with rising IGFBP-3 concentrations.

#### Effect of IGFBP-3 on HUVEC apoptosis

**Fig. 6:**
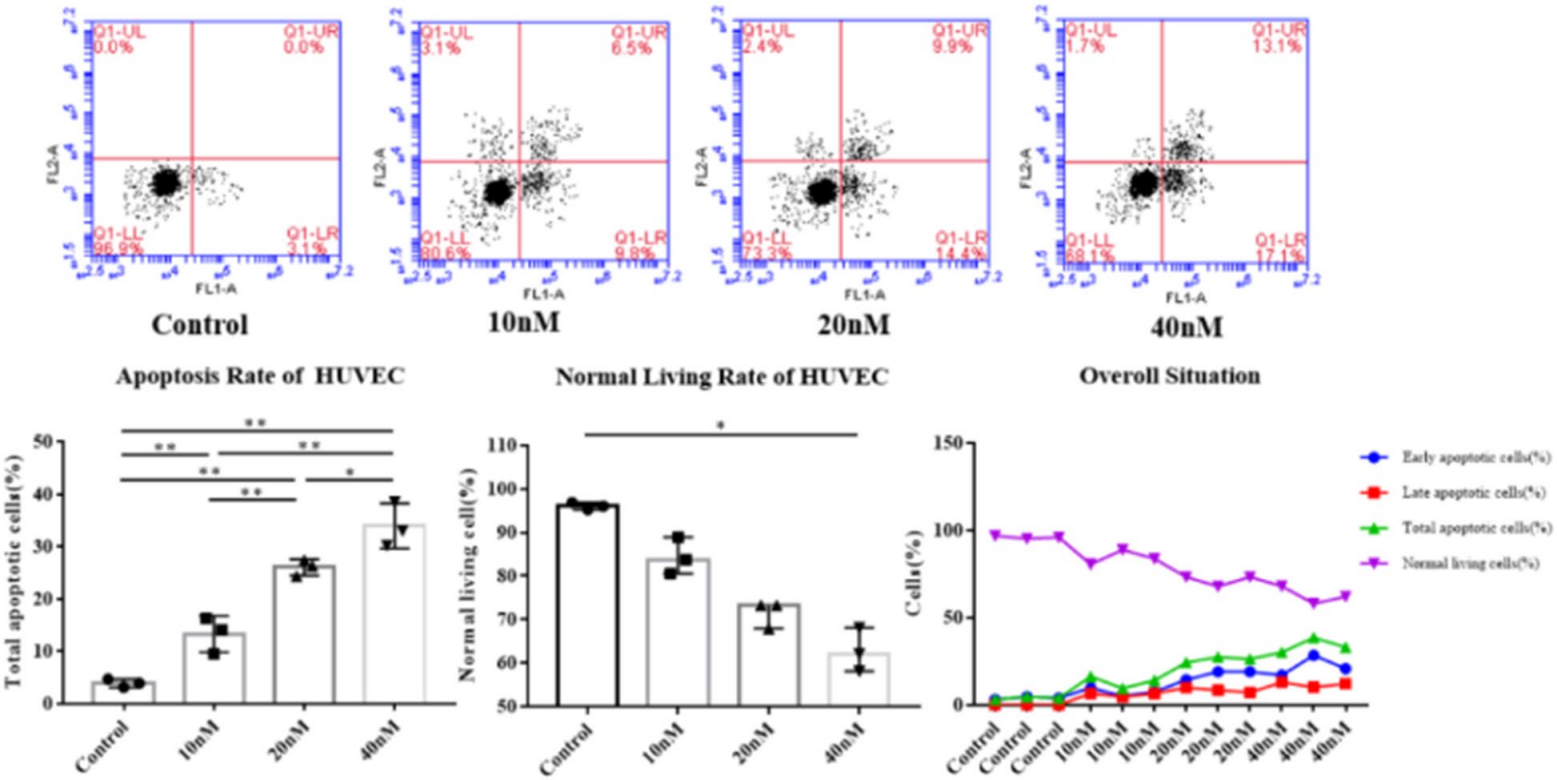
Comparison of apoptosis rate and normal living rate of HUVEC in each group (^*^P < 0.05, ^**^P < 0.01)

Table 4 and Fig. 6 indicate that the total apoptosis rate (%) of HUVEC cultured in different concentrations of IGFBP-3 was 3.90 ± 0.80 in the control group, 13.30 ± 3.47 in the 10 nM group, 26.00 ± 1.57 in the 20 nM group, and 33.93 ± 4.28 in the 40 nM group. These results confirm that there were significant differences among the four groups (*P* < 0.01).

The total apoptosis rate of the 40 nM group was higher than that of the control group (*P* < 0.01), 10 nM group (*P* < 0.01) and 20 nM group (*P* < 0.05). Besides, the apoptosis rate of the 20 nM group was significantly higher than that of the control and 10 nM groups (both *P* < 0.01). Also, the apoptosis rate of the 10 nM group was higher than the control group (*P* < 0.01), with statistically significant differences between each pair of groups.

The normal living cell rates (%) of HUVEC were 96.10 (95.70, 96.5) in the control group, 83.8 (82.20, 86.35) in the 10 nM group, 73.30 (70.60, 73.3) in the 20 nM group, and 62.1 (60.10, 65.10) in the 40 nM group, with significant differences among the four groups (*P* < 0.01). Comparisons between each pair of groups did not exhibit significant differences (*P* > 0.05), except that the rate of the 40 nM group was noticeably lower than the control group (*P* < 0.05).

In conclusion, IGFBP-3 increases the total apoptosis rate of HUVEC, which rises along with IGFBP-3 concentrations. In contrast, IGFBP-3 reduces the normal living cell rate of HUVEC to a certain extent.

## Discussion

As a rare but serious complication of pregnancy, HELLP syndrome involves various systems and multiple organs, causing great harm to pregnant mothers and infants [62]. The vast majority of HELLP cases develop from PE, but they are far more dangerous [2, 8, 54]. From a clinical point of view, early diagnosis and timely treatment are extremely effective in reducing the risks of HELLP syndrome to mothers and perinatal infants. Unfortunately, there are still no efficient clinical predictive or diagnostic markers, nor any effective targeted therapies for HELLP syndrome.

To discover novel and efficient serological markers of HELLP syndrome, as well as therapeutic targets for it, we conducted protein microarray analysis for the serum of patients from a previous study. Through bioinformatics analysis, we gradually focused on IGFBP-3 [41], which had not been reported in the field of HELLP syndrome.

IGFBP is a superfamily of proteins with highly specific binding to IGF, including IGFBP-1 to IGFBP-6 [63]. As a member of the IGFBP family, IGFBP-3 is abundant in serum and most of the IGF-1 in the body exists in a complex form with it. Human IGFBP-3is mainly produced in the liver, but also other tissue, such as the placenta, uterus, and kidneys [64]. The gene encoding human IGFBP-3 is about 8.9 KB in size, is located on the short arm of Chromosome 7, and contains five exons. The members of the IGFBPs are generally similar in structure, and the total length of the peptide chain contains 216–289 amino acids (average: 260) and the molecular weight is between 23-31 kDa. They consist of three parts: the central variable region, N-terminal and C-terminal conserved domain [42]. The N-terminal and C-terminal of each IGFBP both contain a large number of structurally similar cysteine residues, besides, N-terminal domain of IGFBP-3 contains the IGF binding sites that are closely related to IGFBP-3 function [42, 64]. The central region of IGFBP-3 contains 95 amino acids, and it is structurally different from other members with less than 15% similarity. It consists of three glycosylation sites (ASN-89, -109, -172) as well as multiple phosphorylation sites and other post-translational modification sites. The C-terminal domain of IGFBP-3 also has several functional binding sites, such as the IGF-1 binding site [65]. IGFBP-3 involves a variety of functions, several of which are related to the regulation of IGF and are known as IGF-dependent functions. This refers to a series of IGF regulatory functions concerning transport in vito, interaction with receptors and tissue specificity [66]. Additionally, other functions are independent of IGF, known as IGF-independent functions [67].

All clinical subjects in this study were hospitalized pregnant women. According to a comparison of the clinical baseline data and laboratory indicators of them, we confirmed that the subjects represented the main clinical characteristics of HELLP syndrome, PE, and normal healthy pregnant women, respectively. By ELISA, we found that the level of IGFBP-3 in maternal and umbilical blood of HELLP syndrome patients was higher than in normal healthy pregnant women and PE patients, which was consistent with our previous results from protein microarray analysis [41]. Under the superficial implantation of villous trophoblast cells, uterine spiral artery recasting is limited. As a result, vasospasm and contraction of blood vessels cause relative ischemia and hypoxia of the placenta as well as systemic vascular endothelial cells [53]. In this environment, the placenta releases a variety of placenta-derived factors, including IGFBP-3 [54]. When a large amount of IGFBP-3 is released into the circulation, its balance in the body is disrupted and the normal physiological regulation function is also affected. In this study, we found that IGFBP-3 level in the maternal and umbilical blood of PE patients was higher than that of healthy pregnant women, which confirms the above inference to a certain extent. In the state of placental hypoxia in PE itself, the amount of IGFBP-3 produced by the placenta increases. It is released into the blood circulation, leading to higher IGFBP-3 level of PE patients than in healthy pregnant women. As a severe complication of PE, the placental hypoxia of HELLP syndrome is far more intense than that of PE, so the amount of IGFBP -3 released is more greater. Consequently, the level of IGFBP-3 in HELLP syndrome patients is higher than in PE patients. IGFBP-3 affects the transduction of corresponding molecular signaling pathways in the body and the function of its downstream core molecules through IGF-dependent and IGF-independent pathways. It then aggravates vascular spasms by reducing the production of NO, thereby exacerbating the degree of hypoxia of endothelial cells and promoting the release of more inflammatory mediators. In particular, this process can lead to the damage and dysfunction of vascular endothelial cells and hepatic sinusoidal endothelial cells, as well as intensify cell apoptosis, gradually promoting abnormal liver function, microvascular hemolysis, and the initiation of platelet activation and consumption. Thus, it may ultimately lead to the development of PE into HELLP syndrome.

Several studies have indicated that IGFBP-3 promotes cell apoptosis, although these studies were mostly related to tumors [68]. Nickerson et al. discovered from in vitro cell experiments that the addition of IGFBP-3 significantly increased the apoptosis rate of breast cancer MCF7 cells [67, 69, 70]. In this study, we confirmed that IGFBP-3 increased the apoptosis rate of HLSEC and HUVEC cells while reducing the ratio of normal living cells. These effects were especially prominent in HLSEC cells. This correlates closely with the incidence of HELLP syndrome, which affects the normal function of the liver and vascular endothelium, thus making it an important link in the pathogenesis of the disease. Therefore, it can be inferred that IGFBP-3 is involved in the pathophysiology of HELLP syndrome by increasing apoptosis and affecting the normal function of the liver and vascular endothelium.

IGF-1 is related to the regulation of various biological functions of the body. When IGF-1 binds to IGF-1R, it stimulates a cascade of molecular signals in the cell through insulin receptor substrate (IRS) and Src homologs. This subsequently regulates the transduction of downstream pathways such as phosphatidylinositol 3-kinase (PI3K)/protein kinase B (Akt), thereby affecting cell proliferation and apoptosis [71]. It is widely known that the PI3K/Akt pathway is involved in the process of cell proliferation, inhibition of cell apoptosis, inflammatory response, etc. [72, 73]. Phosphorylated Akt inhibits cell apoptosis by regulating the expression of apoptosis regulatory proteins, while it also mediates the phosphorylation of endothelial nitricoxide synthase (eNOS). This in turn promotes the production of NO and plays a role in vasodilation, as well as inhibiting the adhesion of white blood cells and platelets to the vascular wall, providing an anti-inflammatory effect [22, 74, 75]. In animal studies, the dysfunction of mouse aortic endothelial cells was alleviated by activation of the PI3K/Akt/eNOS signaling pathways [76]. Under normal circumstances, IGF-1 and IGFBP-3 exist in complex forms in the circulation, which prevents IGF-1 from binding to its receptor in the extracellular environment and inhibits its biological activity. However, when IGFBP-3 is hydrolyzed by protease, free IGF-1 increases and binds to IGF-1R, which activates the downstream PI3K/Akt signaling pathway, phosphorylates Akt, and increases the production of NO. This promotes the processes of anti-apoptosis, anti-inflammation, and vasodilation.

According to the results of this study, it is speculated that more IGFBP-3 is produced by the placenta under severe hypoxia. It combines with the IGF-1 in the circulation, resulting in a reduction of free IGF-1 and further inhibiting the binding of IGF-1 and IGF-1R. This affects the downstream transduction of IGF-1 in the PI3K/Akt pathway, blocking the phosphorylation of Akt and activation of the PI3K/Akt/eNOS pathway [66]. As a result, the release of NO is reduced, thereby weakening the anti-apoptosis and anti-inflammatory effects. Besides, the previously contractile blood vessel spasms further exacerbate the hypoxia of cells and increase the release of inflammatory mediators with hepatotoxicity, such as IL-1, IL-6, and TNF-α. This results in further damage to vascular endothelial cells and hepatic sinusoidal endothelial cells, as well as increased apoptosis and a decreased number of normal functioning cells. The above process is probably a key link in the pathological mechanism of HELLP syndrome [67]. In this study, we found that IGF-1 levels in the maternal and umbilical blood of patients with HELLP syndrome were lower than those of healthy pregnant women. Besides, correlation analysis revealed that the levels of IGF-1 were moderately correlated with IGFBP-3. This result verifies our inference above.

In addition to IGF-dependent pathways, IGFBP-3 may function in this disease through IGF-independent pathways. It has been assumed that IGFBP-3 may play an important role in the regulation of the TGF-β1/Smad3 signaling pathway. After ischemia and hypoxia, the production of reactive oxygen species, neutrophils, and complement activation may lead to apoptosis, inflammation, and structural damage of cells and tissue [77–79]. In this process, the transforming growth factor-β (TGF-β)/Smad pathway plays a crucial role. TGF-β is a superfamily composed of multiple cytokines, members of which contribute to physiological processes such as cell differentiation and growth regulation. Type II receptors on the cell surface are autophosphorylated after binding with activated TGF-β1, which then further phosphorylates type I receptors and activate it. The Smad protein family is the substrate of the TGF-β1 receptor and is a mediator of TGF-β1 signal transmission from outside the cells to the nucleus. It participates in the regulation of cell proliferation, transformation, secretion, and apoptosis. Activated TGF-β1 type I receptor promotes the transformation of Smad3 into active phosphorylated Smad3 (pSmad3), which then regulate the expression of target proteins [80]. Studies have confirmed that activation of the TGF-β1/Smad3 pathway plays a role in lesions of these tissues by aggravating oxidative stress, cell damage, andthe inflammatory response of cells and tissue, while simultaneously promoting cell apoptosis, thus induced the damage of organs such as the heart and liver [23, 81–84].

Previous studies have shown that IGFBP-3 activates the TGF-β1/Smad3 pathway by binding to the TGF-β receptor. This provokes damage to cells and tissue by stimulating inflammatory response, while also promoting the apoptosis of cells [23, 81, 82]. Under the severe circumstances of placental hypoxia, the large amount of IGFBP-3 released will excessively bind to the TGF-β receptor and continuously activate the TGF-β1/Smad3 pathway, thereby promoting Smad3 phosphorylation and stimulating the release of proinflammatory mediators. This process also provokes an inflammatory response and the injury and apoptosis of vascular endothelial cells and hepatic sinusoidal endothelial cells under hypoxia, promoting the development of HELLP syndrome [83, 84]. In this study, we established that the levels of TGF-β1 in maternal and umbilical blood of patients with HELLP syndrome were significantly higher than those of healthy pregnant women. Besides, there was a positive linear correlation between TGF-β1 and IGFBP-3, supporting the hypothesis that IGFBP-3 may function through the activation of the TGF-β1/Smad3 pathway to a certain extent.

Additionally, various studies have shown that the expression of VEGF is considerably down-regulated in the serum and placental tissue of patients with PE and HELLP syndrome [85–88]. An in vitro model of PE revealed that VEGF overexpression significantly increased the levels of eNOS and NO while inhibiting the inflammatory response induced by TNF-α [89]. It has also been reported that IGFBP-3 inhibits the formation of vascular networks in human umbilical vein endothelial cells stimulated by VEGF [90]. Since IGFBP-3 has an inhibitory effect on VEGF, when the expression of IGFBP-3 increases in vivo under hypoxia, it may reduce the expression of VEGF in serum, which further affects the VEGF/eNOS/NO pathway and limits NO production. Therefore, vasoconstriction becomes more severe under hypoxia, and the resistance to inflammation weakens. After the intensification of the inflammatory reaction, damage to vascular endothelial cells and hepatic sinusoidal endothelial cells intensifies, thereby promoting the pathophysiological changes in HELLP syndrome. In this study, we found that levels of VEGF in the maternal and umbilical blood of HELLP syndrome and PE patients were lower than those of healthy pregnant women, which was consistent with previous studies. It confirmed our deduction that IGFBP-3 acts on HELLP syndrome by affecting the VEGF/eNOS/NO pathway. Moreover, the moderate inverse correlation between maternal VEGF levels and IGFBP-3 was consistent with the hypothesis that IGFBP-3 plays a role in HELLP syndrome by inhibiting VEGF.

Through analysis, we established that the level of IGFBP-3 in maternal blood was moderately correlated with PLT/ALT/AST/LDH in maternal blood, while its correlation with PLT/ALT/LDH was linear. This suggests that IGFBP-3 may be associated with the abnormal degree of diagnostic indicators of HELLP syndrome, and also somewhat reflects the severity of the disease, which causes concern among clinicians. At present, the diagnostic indicators of HELLP syndrome are very limited, and there is a lack of efficient serum markers for the prediction of it. Also, there is nota clear therapeutic target for treatment once the disease occurs. Since IGFBP-3levelin maternal blood is associated with the severity of HELLP syndrome, it has certain practical clinical prospects and may be employed as a supplementary indicator for the diagnosis and evaluation of this disease in future studies. It is also worth exploring whether the inhibition of its expression can alleviate HELLP syndrome.

In summary, this study was the first to investigate the correlation between IGFBP-3 and HELLP syndrome. We determined that IGFBP-3 expression was significantly higher in patients with HELLP syndrome, which may play a crucial role in the pathophysiological changes of this disease. However, our study only preliminary verified the association between IGFBP-3 and HELLP syndrome, being certain limitations. Firstly, we have only demonstrated elevated IGFBP-3 level in clinical specimens from patients who have already developed HELLP syndrome. Hence, those pregnant women with elevated serum IGFBP-3 before diagnosed as HELLP syndrome, especially PE patients, whether or not their risk of developing HELLP syndrome will be correspondingly higher? This problem is very worthy of study. At present, we only conducted a retrospective test. If conditions permit, we will expand the sample size to test the serum IGFBP-3 level of normal pregnant women in the first or second trimester, and observe whether the incidence of HELLP syndrome in the third trimester will increase in those with higher IGFBP-3 level, as well as some complications such as Fetal growth restriction(FGR) and placental abruption. Secondly, more studies are required to further explore whether IGFBP-3 affects the pathological mechanism of HELLP syndrome through the regulation of core molecules in the above-mentioned pathways. Due to the limitation of the serum amount collected, only IGF-1, TGF-β1 and VEGF were measured preferentially, while some inflammatory factors were not measured at the same time. This shortcoming should be compensated accordingly in subsequent animal and cell experiments. Also, in the future researches, we need to collect more clinical samples, either sera or placenta tissues, to improve the limitations in this study. Thirdly, our previous study has revealed that many differential proteins maybe related to HELLP syndrome. In the mechanism of pathophysiological changes of the disease, the synergistic interaction of multiple proteins may be the factor that really plays a role. Therefore, IGFBP-3 may be important in the pathological mechanism of HELLP syndrome, but it does not necessarily play an effect alone. It may be interactions with other proteins contribute to the progression of HELLP syndrome. However, it is impossible to study all the differential proteins at the same time, so we focused on IGFBP-3 at first, and will explore its interaction with other differential proteins in the pathophysiological changes of HELLP syndrome after achieving phased results in future.

In conclusion, we hope that more studies in the future will clarify whether IGFBP-3 can act as a therapeutic target or a new serum marker for the diagnosis, prediction, and prognosis of this disease. This will contribute to reducing the harm of HELLP syndrome to maternal and infant health.

## Data Availability

The datasets used during the current study are available from the corresponding author on reasonable request.
